# Causes of death in vertically infected paediatric HIV-seropositives- Karnataka experience

**DOI:** 10.1186/1742-4690-9-S1-P144

**Published:** 2012-05-25

**Authors:** Suresh Shastri, Bharat Rewari, Pavithra Boregowda

**Affiliations:** 1 Karnataka State Aids Prevention Society, Bangalore, India

## Background

Children represent a population at higher risk of HIV-1 infection and AIDS-related death. Tuberculosis is a common cause of severe lung disease and death in children infected with HIV, particularly those living in areas of high tuberculosis prevalence. We investigated the causes of death in HIV-infected paediatric patients.

## Methodology

A retrospective survey conducted in 29 ART centres in Karnataka, India. Medical records of all deaths that occurred between January and September 2011 amongst paediatric patients were reviewed. Immediate and underlying causes of death were described.

## Results

Ninety-seven deaths occurred between January-September 2011. 55% of the deceased were males. The mean duration of survival on antiretroviral therapy was 36 weeks. Median age at time of death was 10 years (range 1-19) median CD4 count was 210 cells/µl (7-2500); 57% had CD4 cell count <250 cells/µl. In all, 64 causes of death were reported. In 44% (28/64), the causes were WHO clinical stage IV AIDS-defining illness, 36% (23/64) and 2% (1/64) were stage III and stage II conditions, respectively. Adverse effects to anti retrovirals were noted in 6% (4/64) of patients. Multiple causes were also reported in 6% (4/64). Other immediate causes of death were cardio respiratory arrest (2), suicide (1) and intra-cerebral haemorrhage (1). Infections were noted in 22% (14/64) patients. 43% (23/64) patients had tuberculosis at the time of death. Patients dying from AIDS-related events were more often men (17 out of 31).

## Conclusion

Although antiretroviral therapy has substantially and dramatically decreased AIDS-related opportunistic infections (OIs) and deaths, prevention and management of OIs remain critical components of care for HIV-infected children.

